# An Anatomical Description of the Diseases of the Organs of Circulation and Respiration

**Published:** 1846-07

**Authors:** 


An Anatomical Description of the Diseases of the Organs of Circulation and Respiration. By *Chas. Ewald Hasse*, M.D. Professor of Pathology, &c. at Zurich. Translated and Edited for the Sydenham Society, by *W. E. Swaine*, M.D. London, 1846. 8vo. pp. 400.

Elements of Pathological Anatomy. Illustrated by colored Engravings, &c. By *Samuel D. Gross*, M.D., Professor of Surgery in the Medical Institute of Louisville. 2nd Edition. Philadelphia, 1845. Royal 8vo. pp. 822.

Inasmuch as it is our vocation to bring before the notice of our brethren whatever is of interest and importance in connection with the medical literature of the day, we have always considered that our readers may fairly claim from us some notice of works which, though not published, are issued by a society established expressly for the purpose of benefitting the profession by the diffusion of sound medical literature. We do not, however, consider it our duty, nor is it our intention, to criticize the proceedings of the Council of the Sydenham Society—that they have a delicate and difficult task to perform all must admit, and few will be disposed to refuse them full credit for an anxious desire conscientiously to discharge the onerous duties that have been confided to them. We hail with heartfelt satisfaction the present flourishing state of so important a Society.

The present work we have carefully examined, and hesitate not to express our conviction of its great intrinsic value and practical interest. Although our continental neighbours have led the way in the study of Pathological Anatomy, there has of late been no lack of successful labourers in this department of medical science among ourselves. But hitherto no work of a precisely similar character to the present has issued from the British press. None which appears so calculated to make morbid anatomy subservient to its true and only really useful object, the history of disease—a knowledge of which is essential to all rational treatment. Professor Hasse's work is not a mere dry detail of the morbid appearances presented by the various tissues and organs of the body, nor, as his editor states, a mere descriptive catalogue of curiosities in morbid anatomy. The author's aim has been to make the actual knowledge of pathological anatomy subservient to an "anatomical history of disease," and thus to render it of practical utility at the bed-side.

The advantages which Professor Hasse has possessed for the preparation of such a work as the present, appear to have been considerable, and of these he has manifestly availed himself to the utmost. As a diligent student in the hospitals of Paris and Vienna, and subsequently as clinical assistant to Professor Carus, and pathological prosector in the principal hospital at Leipzig, he possessed the means of observing and collecting materials for himself, whilst, at the same time, he was forming that "pathological collection," which, under his auspices, has grown into a most interesting and valuable Museum. The present treatise, therefore, differs essentially from what is commonly called a compilation. For although he has "not relied solely on his own investigations, but has largely availed himself of facts recorded by others," he has been chary in making use of other men's experience. This, perhaps, may account for some apparent deficiencies. The estimation in which the book is held in Germany is sufficiently attested by the fact that since its publication the author has had the offer of the chair of Clinical Medicine in five Universities, and now holds that vacated by Professor Schöenlein at Zurich.

Before proceeding to give our readers an account of the work itself, we have only to add, that the author's reputation does not appear likely to suffer at the hands of his English translator and editor. The translation is, as a whole, very well executed. And although occasionally the version would have been improved by a less literal rendering, and further use of the pruning knife, Dr. Swaine has acquitted himself most creditably and honourably in what appears, from his preface, to have been a labour of love. The getting up of the book, like that of most of the Sydenham Society's publications, is beautiful.

The allied work, of which the title stands at the head of this article, is of a very different character from that of Professor Hasse. It is a ponderous tome of between 8 and 900 pages royal octavo, of transatlantic origin, emanating from one of the American Universities. As it has arrived at a second edition in the course of a few years, it may be presumed to be held in some estimation by our American brethren. From the author's preface it would indeed appear that it is the only system of pathological anatomy of American origin, and that this branch of medical science has hitherto been very much neglected on the other side of the Atlantic. We doubt not, therefore, that the author has conferred an important service on his countrymen by the compilation of this work. As, however, it is little else than a tolerably well executed compilation, having few or no pretensions to originality, either of design or execution, it comes before us under very disadvantageous circumstances, after the perusal of the delightful and original work of Hasse, which is beautifully written and marked throughout by a philosophical spirit and critical acumen that cannot fail to strike and captivate as well as benefit every careful reader. Moreover, there is in the work of the American Professor much that we cannot but consider as irrelevant matter. Lengthened introductions on the normal anatomy of the organs and tissues prefixed to each section treating of their morbid conditions are certainly out of place, and needlessly swell the size of the volume. Nor is it consonant with the title of the work, "Elements of Pathological Anatomy," to introduce disquisitions on such subjects as the general doctrines of Inflammation, to say nothing of many others which are rather physiological than pathological. We hold it to be a duty in these book-making days to protest against this encyclopædical method of treating all subjects. With these preliminary remarks, we proceed to give our readers an account of the two works. We shall, however, take Professor Hasse as our guide, and our remarks will principally have reference to his work.

The present volume of Hasse's Pathology treats of Diseases of the Organs of Circulation and Respiration only, and is divided into two Parts. The first consists of five Chapters, comprising the diseases—1, of the Lymphatic Vessels and Glands—2, of the Veins—3, of the Arteries—4, Heterologous Formations in the Circulating Organs—5, Diseases of the Heart.

Having pointed out the general participation of the lymphatics in all inflammatory diseases, and that the inflammatory process is originally developed in the contiguous cellular tissue, and is thence communicated to the walls of the canals—that in consequence of the check presented by the glands, the diffusion of morbid products is much less frequent by the lymphatics than by the veins, that it is not till the irritating matters have reached the lymphatic glands, and have undergone organic assimilation that inflammatory re-action is established, Hasse proceeds to demonstrate how the lymphatic vessels are influenced by the vitiated condition of their contents. The first condition requisite for that septic inflammation which attends the absorption of morbid matters, is asserted to be a liability to transudation of the morbid contents through the parietes of the lymphatic vessels. This species of exosmosis, he thinks, is induced "partly by the immediate action of the irritative matter on the membranes of the vessels, partly by the clogging and distension of the latter, in proportion as the lymphatic glands become less permeable to the affluent lymph." Thus we find the surrounding textures saturated with the transuding fluid, at first limpid, afterwards turbid and becoming converted into pus, in the midst of which the lymphatics are found, as thin knotty cords, with thickened coats, and their internal membrane no longer smooth.

As the suppuration spreads, the lymphatic vessels are destroyed, and circumscribed abscesses are formed. In this way inflammation extends from one group of glands to another, and occasionally to the trunk of the lymphatics, even into the veins, when we have all the general consequences of the admixture of pus with the circulating current.

The lymphatic glands frequently become inflamed, either separately, or in conjunction with the lymphatic vessels. The affection may be either acute or chronic, and of a traumatic, rheumatic, or septic nature. It may occur sympathetically from disease in neighbouring organs, or from pseudo-formations, *e. g.* tubercle within the glands. Having briefly described the progress and effects of inflammatory action in the glands, he observes that inflammation of the lymphatic vessels and glands is a sequence of very various diseases, but especially of such as result from the introduction of noxious matters, or which represent some constitutional cachexia. The following are mentioned as the most important ways in which the lymphatic system is involved—from "poisoned wounds," "the plague," "typhus," "syphilis," "porrigo," and "elephantiasis." The inflammation of the occipital and other glands in connection with extensive porriginous inflammation of the scalp, is admitted by Hasse to be of doubtful specific character. But, for our own part, we see no reason whatever for assuming that there is anything specific in the character of such glandular inflammation. It may be seen in connexion with any extensive cutaneous disease of the head. It is true that such glandular affections may, as in the case of buboes, be of two different kinds, the one resulting from the direct reception of the virus, the other of purely inflammatory origin. The pus of the former, according to Ricord, is alone capable of transmitting syphilis.

Of dilatation of the lymphatics, whether partial and assuming the appearance of hydatids, or general, neither Hasse nor the American Professor is prepared to offer any explanation, except in those cases where it is manifestly the result of mechanical causes.

The first Section of the 2nd Chapter of Hasse's work is devoted to Phlebitis, which he very properly adduces as an example of the important services rendered to practical medicine by the study of pathological anatomy. The true nature of various, so termed, malignant intermittents, typhoid conditions, puerperal diseases, and the like, has been revealed by the discoveries connected with phlebitis. Inflammation of veins is naturally considered under two heads, the one comprising the purely local, primary phenomena, the other the general secondary consequences, diffused throughout the whole system. The description which Hasse gives of the appearances presented by an inflamed vein is carefully drawn, and is a good specimen of the author's style. The discolouration of the lining membrane of an inflamed vein is said to differ in no respect from the redness of imbibition, except in the mottled alternations of its various shades. But the surrounding cellular tissue early "exhibits an incipient infiltration of a faint-red serous fluid, together with a dense network of delicate little vessels, which, in the larger veins, are distinctly seen to extend to the cellular coat of the vessel. A double consequence now appears to ensue, —and in most instances very rapidly: namely, deposition of an inflammatory product within the canal of the vein, and a change in the blood itself. For, as the morbid blood vitiates the membranes of the vein, producing inflammation, so, in like manner, these membranes, when once inflamed, exercise a reflex action upon the blood; the first sign of which is, probably, the inflammatory redness of imbibition already described. The further changes consist in the formation of fibrinous deposit, at first loosely connected with the internal membrane of the vein by means of a tenacious mucus-like substance, but which subsequently adheres more and more firmly through the medium, as it were, of cellular tissue." P. 12.

After describing the subsequent processes which follow the formation of the plug by which the vein is, for a time at least, obliterated, our author lays great stress on the importance of the changes taking place in the surrounding cellular tissue. This becomes condensed, assumes a brawn-like character, and is intimately connected with the external coat of the vein, which is thus kept permanently distended, so that when cut through its calibre remains patent, like that of an artery. In dilatation of the veins a similar state of things exist, and the danger of pus passing into the circulation after the excision of hæmorrhoidal tumours, &c. will be proportionate to the number of vessels in this state of permanent patency within the wound.

Although the question, how pus is formed within inflamed veins, belongs, strictly speaking, to general pathology, its importance, in connexion with the topics under discussion, has induced the author to give a brief account of the present state of our knowledge on this subject, and he appears to decide for the view which supposes the pus-globules to be the result of changes in the formation of epithelium-cells, and not modified blood-corpuscles. The latter, however, are altered in form, and cling to one another and to the sides of the vessels. The mingling of pus with the blood causes its coagulation, the contents, moreover, being unceasingly propelled towards the heart, the more or less solid products of the inflammation are conveyed beyond the original site of the morbid action; so that we cannot always decide that the part of a vein at which we may happen to find a pus-coagulum is the true seat of the disease, for this is frequently remote and difficult to discover. The pus, however, more usually becomes isolated by the coagulation of the blood, and the consequent impediment to its circulation, and this constitutes what Cruveilhier has termed sequestration of veins. It is only when this sequestration is prevented, or is imperfect, and the pus and softened fibrin pass into the current of the circulation, that we have the serious constitutional typhoid phænomena. The further organic changes consequent on this morbid impregnation of the blood are divisible into such as occasion a stagnation and interruption of the sanguineous current in the central portions of the vascular system, and such as have their seat in the capillary system. But that something beyond the mere presence of morbid matter in the blood is necessary to account for the coagulation and interruption of the current of the blood is manifest from the observations of Glüge, viz. that pus-globules are found in the blood contained within the heart in phthisis, and yet little or no coagulation occurs. The occurrence of lobular abscesses, or purulent deposits in the lungs, &c., Hasse does not hesitate to ascribe to phlebitis, which, in many instances of external injuries and operations, may readily be overlooked. He admits, however, that sometimes there may be direct absorption through the open mouths of wounded or ulcerated capillaries. But although we must, with our author, admit that no such thing as absorption of unaltered pus through the sides of the capillaries takes place, yet, from the experiments of D'Arcet and Sedillot, there is every reason to believe, that some of the principles of putrid or altered pus may thus be absorbed, and, moreover, that the occurrence of typhoid symptoms may be dependent, not so much on the mere existence of pus in the blood and the consequent purulent deposits, as on the altered or putrid character of the disintegrated pus-globules, or of the serous and albuminous principles of the pus. Neither Hasse nor Gross, however, makes any distinct reference to this, which we consider an important part of the question. It appears also highly probable, from D'Arcet's experiments, that by exposure to the influence of oxygen in the lungs, pus, which may have been conveyed thither in substance, may undergo there those changes on which the occurrence of typhoid symptoms depends.

There is, at all events, an important distinction to be drawn between those cases in which we merely have lobular abscesses from mechanical impediments to the capillary circulation in the lungs or liver, and those in which, from the first, there is marked depression of the system, and rapid supervention of typhoid symptoms. But we have occupied so much space with this subject, that we must pass over the remainder of this and the next section, including puerperal phlebitis, phlegmasia dolens, and obliteration of veins, merely calling our reader's attention to an important variety of phlebitis, viz. inflammation of the sinuses of the dura mater. The anatomical characters of this resemble those of ordinary phlebitis. It is mostly a secondary affection, but it may occur as a primary disease. Death ensues very speedily, Hasse observes, amid the phænomena of functional disturbance of the brain. A very interesting case of primary inflammation of the right cavernous sinus has however lately been described by M. Breschet of La Charité, and is mentioned in a recent report on Pathology and Practical Medicine, by Dr. J. R. Bennett. The primary symptoms were, shivering and intense cephalalgia occurring after exposure to wet and cold. These symptoms were succeeded by pain in the eyes, photophobia, pain in the right shoulder and neck, and over the orbits, with œdema of the right conjunctiva. Delirium, dilated pupils, and typhoid symptoms preceded death, which did not occur till the 31st day from the beginning of the illness.

To dilatation of veins, which Gross dismisses in a single short meagre paragraph, Hasse devotes an entire section comprising 20 pages of his second chapter. Phlebectasis, or Dilatation of Veins, he believes to be one of the most frequent morbid changes of structure occurring within the human body. There are three varieties of disease which, anatomically speaking, are referrible to dilatation of veins, varicose veins, properly so called, varicocele, and hæmorrhoids. The chief and common source of all these varieties is referred to a peculiar habit of body, a morbid predominance of the venous system. Exceptions, however, may, we think, be taken to this view, as well as to that which admits all these varieties to possess an hereditary character. No doubt, in many instances, there is a peculiar disposition to dilatation of the veins, without, however, much evidence of this being referrible to any predominance of the venous system. It is remarkable, as Hasse states, that in men, the subjects of varices of the leg, the disease usually arises from the trunk or the principal branches of the saphena, whilst, in women, it commences in the minutest cutaneous twigs. An excellent and lucid sketch is given of the attendant anatomical changes and the consequences of varices of the leg, which our limits compel us to pass over.

*All* hæmorrhoidal tumours are considered by Hasse as referrible to dilatation of veins, but Gross admits two varieties, one of which consists in the extravasation of blood into the surrounding cellular tissue from rupture of a hæmorrhoidal vein. Andral, Lobstein, Puchelt, and Hodgson, with other high authorities, coincide with the opinion adopted by Hasse, in support of which both he and Brodie assert that it is in general easy to inject, from the inferior mesenteric artery, piles not in a turgescent state, and that the reason why Chaussier, Recamier, and others found the injection pass merely into the cellular tissue was, that the piles were, at the time, in a state of erethism, or filled with coagula. There is, however, a tumour of a very different character from the true hæmorrhoidal, though often confounded with it, which Gross has well described. "It consists of an effusion of serum in the submucous cellular tissue, in the form of a rounded, or ovoidal elevation, from the volume of a cherry to that of a small hicorey nut; it is of a pale yellowish colour, or nearly perfectly white, almost transparent, glossy, and very much like the vesicle of a common blister. It pits slightly under the finger, and generally attains its full size in ten or fifteen hours. A few delicate straggling vessels, the colour of which beautifully contrasts with that of the other parts, may often be seen, ramifying over its walls, or intersecting its substance. The tumour, although sometimes multiple, is almost always solitary. The disease is strictly analogous to œdema of the vulva or glottis."—*Gross*, p. 625.

The following is Hasse's account of the difference between arteries and veins, in regard to their susceptibility of inflammation and the occurrence of secondary symptoms. After referring to the experiments of Trousseau and Rigot, to shew that only the most powerful irritants injected into arteries will excite inflammation, he says—

"This may be accounted for by the different structural relations of arteries and veins; in the latter every irritant quickly penetrates the internal membrane, and the peculiar, but very thin, felt-like fibres of the so-called middle tunic, so as to reach the cellular coat; in the latter, there is interposed between the internal non-vascular membrane and the cellular coat (rich in blood-vessels and nerves), a pretty strong layer of elastic texture, possessing a very low degree of vitality, so that the cellular coat is endued with all the vegetative and animal activity of the artery. Accordingly, this latter coat is highly susceptible of every irritation, and readily inflames when acted upon by either chemical, dynamical, or mechanical influences." P. 60.

"The secondary symptoms," he observes, "which play so decided a part in phlebitis, are very seldom observed to follow arteritis. This cannot be attributable to death ensuing earlier in the latter than in the former disease; for in phlebitis lobular abscesses frequently form with great rapidity, whilst in some cases of arteritis the fatal issue is deferred until a very late period. The real cause seems to depend upon some peculiarity in the arteries, in virtue of which plastic matter only is deposited within their canals, and inflammation restricted to the adhesive form. Hence, the more solid product of morbid exudation is not under the control of the heart's impulse, and can alone pass into the circulation when the exudation and the coagulated blood, or the fibrin deposited by the blood, are too scanty to close up the arterial caliber completely, or for a sufficient length of time. This peculiarity manifests itself most distinctly in the tying of arteries, after which operation, notwithstanding the long sojourn of a foreign body, there results a firm plug and adhesion, unless from a concurrence of extraordinary circumstances, diffuse suppuration is induced, which then commences in the surrounding parts and in the cellular tunic, and only *indirectly* invades the lining membrane of the artery,—the plug being originally *always* of a purely plastic nature. In simple arteritis the circulation in the involved artery is, in like manner, mechanically arrested, up to the nearest diverging branch, by the product of the inflammation, just as it is from the application of a ligature. Should suppuration ultimately occur even *under these circumstances*, it remains purely local,—shut out from the general current of the circulation, however far it may extend downwards in the direction opposed to that of the heart. Should the purulent softening extend upwards, so as to destroy the closing plug, the blood-stream will find its way back to the inflamed portion. Here, however, the artery is no longer entire, since the suppuration usually begins in the external coat, and in its progress destroys the others. Thus the blood, meeting with no resistance, perforates to a greater or smaller extent the softened membranes. If this happens with an artery to which a ligature had been attached, a fatal hemorrhage necessarily ensues, unless a fresh ligature be instantaneously applied higher up. In spontaneous arteritis occurring in parts previously sound, there ensues either extravasation, or a so-called *diffuse spurious aneurism*, the hemorrhage being restrained by the thickened and indurated cellular texture in the neighbourhood of the ruptured part. Such occurrences sometimes follow external injury; but in spontaneous arteritis they are so rare, that I can call to mind but a single authentic example, namely, the case before cited from Hodgson's work." P. 63.

Spontaneous gangrene in the aged, the gangrena senilis of Pott, is we think correctly considered by Hasse as the result of essentially the same disease as occurs in the earlier periods of life, viz. arteritis, and that the ossification of the arteries is the accidental and remote, rather than the proximate, cause of the gangrene. Gross also adopts the same view. This is a question of great practical importance, to which the attention of the profession has of late been especially called; and it appears to us that the symptoms during life, the comparative results of the old stimulating plan, and of a modified antiphlogistic treatment, together with the appearances observed after death, alike prove the correctness of the view adopted by Hasse. It cannot, however, be doubted that there is considerable pathological diversity in the various cases which have been described under the name of spontaneous gangrene, and that the true senile gangrene connected with degeneracy of the arterial coats presents distinctive features. A little work published a few years ago by Hecker of Stuttgard contains an excellent account of almost all that is known on this subject.

The remaining sections of this chapter are occupied with the subjects of atheromatous disease, and ossification of arteries and aneurisms, of which a very instructive and succinct account is given. A short chapter is devoted to heterologous formations in the circulating organs. "No well authenticated example is known," says our author, "of the arterial membranes being the seat of heterologous growths. Whatever have been described as resembling such, have been either various forms of atheromatous degenerations, or else tumours intimately incorporated with the filamentous sheath. This immunity of the arteries has led to the idea of identifying atheroma with tubercle, a groundless assumption, seeing that the two substances are essentially dissimilar, and also that arterial degeneration occurs but seldom and never to any extent in the tubercular habit."

The veins, on the other hand, it is well known, may become diseased in various ways, and the occurrence of fungoid, or lardaceous growths within the veins has excited much attention.

"Some refer it to the absorption of the heterologous matter by the minutest venous twigs, others to immediate deposition from the blood. The former view is at variance with physical laws. The primitive cells of those growths exceed the blood-globules in size; it is therefore inconceivable that they could traverse, supposing they could enter the closed capillaries, unless indeed the nuclei of the cells be reckoned the germinal principles of disease. Nor will the second view hold good until the proximate elements of fungus can be *proved* to pervade the whole sanguineous mass." P. 108.

That we have not as yet satisfactory proof of the latter theory, of the origin of cancer-cells in the blood, must be admitted, still we think that no satisfactory theory of either the physiology or pathology of malignant diseases can be given, except by the admission that, the primary blastema may and does occasionally germinate within the vessels. This subject has been discussed by Dr. Walshe in his work on Cancer with great acumen, and he appears to have been compelled to come to the same conclusion.

In the fifth chapter, which treats of Disease of the Heart, our author gives due prominence to the various modifications of pericardial inflammation as influencing most materially the ulterior course of the disease and the consequences to which it gives rise. Both in respect of prognosis and treatment it is of much practical importance to be aware of the great difference that exists in the character and amount of effusion which attends pericarditis. The most intractable forms of pericarditis are referable frequently not so much to the violence of the inflammation as to the unorganisable character of the effused matter.

"The products of pericarditis are not always uniform, even in the same subject, but as before stated, the same exudation frequently contains various elements, which, by virtue of the ulterior changes wrought in them, modify the course of the disease in many different ways. This variable composition of the exudation may proceed from alternations in the intensity of the inflammation,—a rapid succession of inflammatory attacks giving rise in turn to serous and sanguineous, —organizable and unorganizable effusion; or else it may proceed from some peculiar ingrained morbid predisposition to engender diseased products. Thus it not unfrequently happens that true tubercle, and even medullary fungus, is developed out of the exuded substances, as seen both by Kolletschka and by myself." P. 116.

The Section treating of Endocarditis is admirably drawn up, and most valuable. We regret that our limits prevent our extracting largely from it. Having indicated the distinctions between the various forms of fibrinous coagula found in the heart's cavities, he thus points out the means of discriminating "between redness of the endocardium from inflammation, and redness from imbibition; as also between polypous formations engendered by exudation, and coagula resulting from the inflammatory irritation of other organs."

"The inflammatory redness is almost always spotted,—pale and dark alternately; in one place more of a violet, in another more of a scarlet colour. The redness of imbibition is, on the contrary, more equable throughout, and darker, perhaps, where the blood makes a prolonged sojourn. The redness of imbibition is therefore almost invariably observed in the following descending order: darkest in the right auricle, paler in the right ventricle, with the exception of the valves of the pulmonary artery, which are as deeply coloured as the auricle; still paler in the left auricle, whilst the left ventricle often retains quite its natural tint, except that the aortal valves are darker; in the great vessels the posterior surface is strikingly dark in comparison with the anterior. Moreover, the endocardium continues smooth and polished, and in all other respects natural, whilst inflamed portions of it assume a dull, velvety appearance, and are sometimes thickened and softened. If besides the polypous coagulum the endocardial surface is invested with a soft, firmly adherent false membrane, the inflammatory state is no longer doubtful. At the valves, particularly the tricuspid and mitral, this membranaceous covering takes the character of soft, hemispherical granulations, capable of increasing in bulk, and in some instances, as shown by Kreysig and Bouillaud, susceptible of organization. Secondly, these polypoid concretions can be regarded as products of endocarditis only when met with on the inner surface of the heart, in the manner before described, or when they cannot be traced to any other morbific condition. Here absence of inflammatory redness is not always a sufficient reason for denying their inflammatory origin,—seeing that the redness is very fugitive, more particularly in intense degrees of inflammation, when the colour of the membrane gradually from a dull and equable brown red becomes paler,—changing to a dirty grayish yellow. Carswell has figured a remarkable case of inflammatory polypus, in which the lining of the heart appears preternaturally pale. This polypus was partly of a grayish, partly of a lively red colour ; it exhibited, both on its surface and in its substance, little drops and stripes of pus, had insinuated itself amongst the columnæ carneæ, and adhered as if glued to the equally pallid mitral valve. Legroux and others contend that the pus is the result of inflammation of the fibrinous layers, within which it is sometimes found to accumulate,—an error palpably refuted by Bouillaud. Admitting the above granulations resulting from exudation to be susceptible of organization, it may still be doubted whether the polypous coagula are so. Otto could never detect in them any vascular development. It is true that they exhibit points and streaks of blood, sometimes extending into their interior, and which might certainly *pass for* the rudiments of organization ; but it is not conceivable that foreign bodies of such dimensions could occupy the central organ of circulation without speedily inducing death. The few authenticated instances of organized polypi leave it problematical whether they were anything more than mere vegetations upon the valves; and vegetations there, as elsewhere, can hardly be reckoned among the immediate products of inflammation."

"The first and most constant change resulting from endocarditis relates to the endocardial membrane. This, after the aforesaid red spots and patches have disappeared, loses its smooth pellucid aspect, becomes relaxed, turgescent, and rough, puts on a grayish and dull appearance, and is capable of being stripped off with comparative ease. Rokitansky states that under these circumstances the endocardium frequently becomes the seat of irregularly-jagged fissures, which forthwith form a depot for a grayish yellow coagulum."

"When the inflammation assails more particularly the apparatus of the auricular valves, a still more frequent occurrence is the rupture of one or more of the papillary tendons. This is particularly common at the mitral valves. The filaments or shreds of such lacerated tendons curl up and form little elevations, invested with fibrinous coagulum and hardened lymph, whilst the valve, previously angular, recedes and acquires a broad raised margin." P. 128-130.

After observing that, according to Bouillaud's views, all defects of the valves, all ossifications,—in a word, all diseases of the heart's apertures, are directly referrible to endocarditis, Hasse very justly says :—

"This view does not, however, fully correspond with facts; for, in the first place, experience shows that the several organic changes above alluded to, affect the left side of the heart far more frequently than the right; whilst, according to Bouillaud, endocarditis is observed with equal frequency on both sides, and with most intensity on the right. In the second place, we meet with those indurations and ossifications, especially after the prime of life, too often to admit their having been universally preceded by endocarditis; in many subjects, indeed, the contrary could be proved. Thirdly, most of those changes present, in the mode of their development, so perfect a similarity to the progress of atheromatous disease of the arteries as to render it more appropriate to class them under this head.

"It cannot be denied, however, that, under propitious circumstances, endocarditis may be productive of thickening of the internal lining of the heart and of the valves, as well as of granulations and ossifications of the latter, though much less frequently than Bouillaud pretends. In fact, ossification, even when met with in young individuals, cannot invariably be ascribed to endocarditis. After careful consideration of all the circumstances, it would appear that the following organic changes arise from either a partial or a general inflammation of the internal surface of the heart: the milk-white or mother-of-pearl coloured patches and thickenings,—perhaps themselves the relics of earlier relaxation, disorganization, and scar-like puckerings,—of the endocardium; secondly, membranaceous deposits on the inner surface of the heart, more especially in the left auricle, and also in the left ventricle, upon the septum and about the origin of the aorta. These have the appearance of elevated, rough, and puckered connecting membranes. They are of a dull yellow tint, and are easily separated. To the same class belong adhesions of the valves amongst each other, and with the heart's parietes, rupture of the tendinous filaments of the auricular valves; and, finally, a state of partial destruction of the valves, with irregular notchings, and fringed appendices of variable length. This has heretofore been observed only in the semilunar valves of the aorta, and twice by myself in the pulmonary artery. Sometimes it is complicated with partial adhesion of the valves together." P. 132.

There are certain secondary phenomena produced by endocarditis, which are but little attended to, and of which Hasse thus speaks:

"They are referrible to the effusion almost always consequent upon that affection, in the various forms and localities already specified. A considerable portion of the effused substances being carried away in the first instance by the circulation, and another portion during the subsequent period of softening, it is obvious that these inflammatory products must, just as in phlebitis, act a subordinate part within the capillary system. The *spleen* and *kidneys* appear particularly liable to such changes. In the spleen, coagulated fibrin is found of some breadth at the periphery, but gradually tapering towards the centre, having mostly a tolerably sharp outline and a brownish-yellow hue. Smaller deposits, of a similar kind, are met with in the cortical substance of the kidney; sometimes even intersecting the papillæ. According as the inflammatory product of the endocardium partakes more of the character of fibrin or of pus, the secondary deposit will be of a more or less consistent kind, and shrivel in the event of recovery,—or else liquefy, and terminate in abscess. Though frequent in the localities above indicated, these secondary phenomena are proportionately rare in other organs, as in the liver for instance. Sometimes, however, they occur in the serous cavities." P. 134.

In the sections on inadequate valves and hypertrophy and dilatation of the heart, will be found an excellent account of these important morbid states and their pathological relations, with full reference to the labours of Kingston, Bizot, and other recent investigators. A just tribute is paid to the value and general correctness of the statements and views of Bizot. On the relative width of the pulmonary artery and aorta, our author says:

"I began, several years ago, to pay attention to the width of the pulmonary artery relatively to that of the aorta. In E. H. Weber's edition of Hildebrandt's Anatomy, and in J. Müller's Physiology, it is asserted that the former is narrower by one third than the latter, and from this statement many physiological inferences have been drawn. Great was my astonishment, however, to find that, on the contrary, the pulmonary artery is somewhat more capacious than the aorta; yet, on repeating the trial in a multiplicity of cases, I invariably arrived at the same results. Whilst I was thus engaged, Bizot's comprehensive Researches were published, and proved far in advance of my own. Nevertheless, whenever I found time I continued my admeasurements upon a somewhat more comprehensive principle and improved method. My results, based upon 122 admeasurements, almost entirely coincide with Bizot's, and are richly suggestive in a pathologieal point of view. It should be stated that the measurements from which the general results are taken were made on subjects who, during life, had betrayed no symptoms of diseased heart." P. 156, *Note.*

There is, we think, frequently a very vague and incorrect notion entertained with regard to the relation between cardiac and hepatic disease. On this subject Hasse observes, "hepatic disorders effect inconsiderable passive dilatation in the instance of fatty heart only; they engender, besides, a thickening, only ascertained by measurement, in the walls of the right auricle and ventricle. Thus, in my tables I find almost invariably in the cases presenting the largest dimensions, short of actual hypertrophy, hepatic disease noted; and it appears that enlargement resulting from chronic inflammation, with or without fatty or wax-like degeneration, bears a much closer relation to diseased heart than cirrhosis and simple fatty degeneration. Liver-disease may, however, be a consequence, as well as a cause of disease of the heart. We rarely meet with disease of the heart, coupled with stagnation of blood in the right chambers and in the veins generally, without hyperæmia, and a flabby condition of the liver. Nay, this stagnation may go so far as to occasion extravasation of blood within the parenchymatous texture (Bouillaud). The so-called nutmeg-liver is a very common sequence of hypertrophy and dilatation of the heart."

The phenomena connected with the respiratory organs, are among the most prominent and well-known symptoms of cardiac disease, but it is important to be aware that the pulmonary congestion is apt to merge in inflammation, and we have met with some cases that have strongly impressed us with the importance of being awake to this tendency, for the complication, it need hardly be said, is important and dangerous, and very apt to be overlooked amid the more common and habitual symptoms with which these deplorable cases are attended. On this point Hasse says—

"In several instances of considerable hypertrophy, I have seen the resulting pulmonary affection merge in inflammation. The inferior lobe, sometimes of the left, sometimes of both lungs, was displaced, shrunken, not crepitant, softened within a greater or smaller range, of a clear reddish brown colour, and of augmented density. On being cut into, the section was tolerably even, presenting only here and there those soft elevations which, in pneumonia, produce the well-known granular aspect; these, when aggregated in distinct groups (which was however seldom the case) were moister, more decidedly gray (if not of a dirty yellow), and much softer than the rest of the texture. While the whole remainder of the lungs was marked by an excess of blood, the portions alluded to contained more or less of a dingy reddish troubled fluid; circumstances all combining to establish a peculiar modification of the second stage of inflammation of the pulmonary substance." P. 167.

There are two forms of fatty heart that are well known, the one in which there is an unusual deposition of fat in the cellular tissue betwixt the serous investment and the muscular substance of the heart, and the other where the fat globules collect not only within the compartments of the subserous cellular tissue, but are deposited within the muscular substance, even between its primitive fibres. There is, however, a perfectly distinct species of fatty heart, occurring almost exclusively in hypertrophied hearts, which exhibit at the same time the remains of earlier endocarditis and carditis. In this form, which was first described by Rokitansky, "the fat does not accumulate in masses, there being no fat-vesicles inclosed within fasciculi of cellular tissue, but is beaded, as it were, in minute microscopic granules, closely interlaced, and imbedded among the primitive fibres of the heart's muscles. Those primitive fibres have lost their transverse striæ; the fibrils are friable, and easily reduced to minute molecules. The whole heart may suffer this species of fatty infiltration, although commonly distinct portions alone are diseased. In the latter case the flesh is here and there found, within a certain sphere, pallid and dull, of a dingy yellow—soft and flaccid; at a more advanced stage these spheres become more numerous, and pass very gradually into the healthy mass; the endocardium has become thin and transparent, rendering the dull aspect of the heart's muscle here and there conspicuous; the trabeculæ, and the papillary muscles are either wholly or partially diseased. In its greatest extent this fatty condition may affect an entire ventricle, more commonly the left, although, when partaking of the hypertrophy, the right ventricle, and consequently, as above stated, the whole heart may become involved. Rokitansky is of opinion that spontaneous rupture of the hypertrophied left ventricle is, in the majority of cases, referrible to this disease of the heart's muscle. In youth this form of fatty heart has been observed, even where there was no hypertrophy, the organ being dilated, probably in consequence of the degeneration. Rokitansky believes that, when in such instances the papillary muscles are diseased, the consequent imperfect tension might occasion insufficiency of the valves."

Passing over the remaining section on cyanosis, we come to the second part of Hasse's work.

The first Chapter of this division of the work treats of Pleurisy. After alluding to the frequency of this form of inflammation, and the influence exerted by the degree of the morbid action on the character of the products, and thus on the whole progress and issue of the disease, he describes the first appearances of inflammation of the pleura as consisting in a congested state of its blood-vessels, which are seen congregated here and there, in dense though delicate nets beneath the still transparent membrane. The redness, at first punctated, gradually spreads till the whole becomes uniform, and the membrane loses its polish. At the same time, the subserous cellular tissue manifests increased vascularity and numerous ecchymoses, the meshes or intervals between the cells being here and there filled with a yellowish, half-fluid, half-gelatinous effusion. This implication of the external cellular tissue is, however, by no means constant, and becomes less and less manifest in proportion as the disease advances. Now, though Hasse makes no allusion to the subject, we are disposed to think that much of the difference of character presented by the effused fluids in the earlier periods of pleurisy depends on the implication or non-implication of the external sub-serous tissues. The statement of Gross regarding the changes presented by the serous membrane in the early stages is meagre in the extreme, and in many respects incorrect. Hasse denies the existence of a dry stage of pleurisy, characterised, as it is believed, by a total suppression of secretion, and says, " I have never encountered this dry stage as described; having always, even at the very outset, found the serous fluid somewhat, however slightly, augmented in quantity, and marked by its deep yellow tinge and its increased consistency. There were likewise almost invariably present those grayish or yellowish points the initial and quickly expanding rudiments of membranaceous effusions." P. 184. Gross, however, adopts the more usual, and, we think, the more correct view, and states that, at first, there is " if not an entire suppression, at least a considerable diminution, of the natural secretion." The truth of the position maintained by Hasse, and many of the French pathologists, he thinks, is by no means established. One of the highest authorities in our own country, Dr. Hodgkin, coincides with Hasse, and states, in his 2nd Lecture on the Serous Membranes, that, when turbidity of the serous effusion and false membranes " are not observed in persons who have died of suspected pleurisy, inflammation of the muscles or cellular membrane must, in most instances, have been mistaken for it," and further he says that, from his own experience, he cannot cite any case in which the inflammation, however remote, had not left some traces of its existence. If the inflammation have been " so rapid that no false membrane has been formed, we find an effusion of serum which, in the majority of cases, is sanguinolent or puriform." Analogy, however, is certainly in favour of a suppression of the ordinary secretions in the early stages of inflammation of a serous membrane.

Having portrayed the subsequent steps in the inflammatory process, Hasse enters on the consideration of the mode in which inflammatory products become organised. He justly observes, that as no one has yet been able to watch the movements of blood currents in inflammatory products during life, cadaveric phænomena are deserving of close investigation.

" With this intent, I have omitted no opportunity of removing false membranes of every description, either alone or together with the subjacent pleura; and, having first washed them carefully in cold water, proceeded at once to spread them on glass plates to dry. As the congested blood-canals in inflamed or newly-organized textures, do not part with their contents to the larger veins, as in healthy parts, I have been able to furnish a series of preparations in which the course of at least the larger of the newly-formed branches is conspicuously shown by a surprisingly delicate distribution of blood-vessels.

" Wherever vessels had formed in the adventitious membranes, they proved to be continuations of the branches ramifying in the serous coat; they penetrated the false membrane at numerous points, and then branched out in a stellate manner, or formed into partly divergent, partly parallel, fascicular groups. Hodgkin describes them similarly. Such was the character of the vascular development, more particularly in the gelatinous form of exudation, which, in this respect, so nearly approaches the normal formative substance, as, after a short period, to display ramifications of vessels precisely similar to the parent ones, and, in all probability, created in the manner witnessed by Döllinger in the embryo of fishes. I have repeatedly and very carefully taken up, upon pieces of glass, detached and floating fragments of the gelatinous product in question, and searched for independent vascular development, but in vain ; and I feel convinced, that if such flakes should ever be found to contain blood-vessels, they must originally have clung to the pleura, and been severed subsequently." P. 195.

In less organisable false membranes, these processes do not take place so readily, and in these, Hasse considers the descriptions given by Laennec and Gendrin to be applicable. Where tubercular matter is effused with the more ordinary products, and does not prove rapidly fatal, it is occasionally met with as a residue of the aggregate morbid product in small scattered masses included between the pleuritic adhesions. These isolated masses are susceptible of farther transformations, which Hasse thus describes.

"Most frequently, a deposition of phosphate of lime takes place little by little, until the whole is converted into a hard earthy concrement, constituting an irregular, rough plate, sometimes separable into two layers, which inclose an intermediate residue of the caseous, atheroma-like mass already alluded to. The plates in question are wont to adhere so firmly both to the parietes of the thorax and to the surface of the lungs as to prevent the two folds of pleura being distinctly recognized. These relations apply to most instances of so-termed ossifications of the pleura, which would seem, even where they form in the pleura itself, or in the cellular tissue external to it, to originate in inflammatory action (Hodgkin). The thin lamellæ occasionally found without any accompaniment of pleuritic adhesions, and the fibro-osseous tumours of the pleura, are obviously referrible to a different source." P. 197.

We do not, however, see wherein lies the proof that these changes are referrible to calcareous transformation of tubercular matter. Under the head of Empyema we have looked in vain for any corroboration of the important observations of Mc Donnell with reference to enlargement of the liver or the discharge of the purulent accumulations by the bronchial membrane, independent of any perforation.

The Chapter on Pneumonia, we are compelled to pass over, with the exception of the following passage, the practical importance of which our own experienee has taught us.

"*Drunkards* are proportionately most prone to the disease, and, what is remarkable, it scarcely manifests itself in them by the usual vital symptoms, although spreading, and advancing to the last stage with astonishing rapidity." P. 216.

The next Chapter, entitled "Diseases of the Lung not necessarily dependent upon or allied to Inflammation," comprises gangrene, œdema, hæmoptysis, apoplexy, and the atelectasis of Jörg. Of hæmoptysis considered as a primary affection, Hasse admits four varieties, simple bronchial hæmorrhage (or genuine hæmoptysis), effusion of blood into the pulmonary vesicles (apoplexy in a restricted sense), hæmorrhage from rupture of the pulmonary texture (pneumorrhagia), and the most fatal form arising from rupture of the large blood-vessels. There can be no doubt that much unnecessary alarm is often occasioned by confounding simple bronchial hemorrhage with that which is symptomatic of incipient tubercular disease, and we fully agree with the following remarks:—

"This kind of hæmoptysis is seldom fatal, unless when complicated with other morbid processes. It presents itself in its simplest and least hazardous form, in very plethoric individuals, during the years of puberty, generally ceasing spontaneously when the thoracic organs have become fully developed. There is more ground for alarm, when it occurs as a forerunner of menstruation, or as the reflex of hemorrhoidal ailment, in which cases the attacks often recur to an exhausting amount. Even here, however, it hardly by itself endangers life, however serious its immediate and remote consequences (dropsy, &c.) Examples are indeed known of these bronchial hemorrhages acting vicariously for the menstrual or the regular hemorrhoidal flux, with greater or less violence, periodically, during a space of thirty or forty years, the patients at last succumbing under some other disease. Under these circumstances there is no real local disease, the lung being merely in a state of congestion, or, more correctly speaking of hyperæmia." P. 242.

The propriety of the term pulmonary apoplexy has been disputed, but Hasse very properly says that, in true apoplexy of the lungs, the effusion of blood into the air-cells, or into the general pulmonary texture, is attended with loss of consciousness through sudden suspension of the main springs of vital action. In this sense, since the time of Hippocrates, the term apoplexy has been as applicable to the lungs as to the brain. Indeed, the prostration of the vital powers, from the first, forms the most striking characteristic of these cases, which are seldom accompanied by hæmoptysis.

The anatomical division of pulmonary apoplexy into that in which the effusion is thrown out into the air-cells, and that in which it is produced by rupture of the pulmonary texture, Hasse thinks is borne out by the etiological relations and by the vital symptoms. In true apoplectic effusions the blood, he believes, is thrown out from the twigs of the pulmonary artery directly into the air-cells of the smaller bronchial ramifications, and then becomes consolidated without any further change. The defined limits of the apoplectic centre are owing to the absence of rupture, the effusion being limited to a set of pulmonary cells, supplied by particular ramifications of vessels; in short, to a single lobule, whose covering separates the diseased from the healthy parts. The well-known connexion of pulmonary apoplexy with hypertrophy of the right ventricle, is certainly corroborative of this view, and against the notion which Hasse, and we believe Rokitansky also, controverted, viz. that the apoplectic clot is formed by the reflux of blood from the bronchia. Moreover, by the theory of Hasse, the dark black colour of the clot is accounted for by the complete exclusion of the air.

Under the head of Atelectasis of the Lungs will be found a very good account of that peculiar pulmonary affection of children, the inflammatory character of which Hasse very successfully controverts. We think, however, that we are as yet scarcely in a condition to affirm with confidence that it is nothing more than imperfectly expanded lung. We cannot find that Gross has more than a passing allusion to this interesting morbid condition.

Diseases of the Air-passages are next considered. Under the head of Catarrh, acute or chronic; we have not found any thing specially deserving of notice. The following remarks, however, are of practical value.

"It is remarkable that senile catarrh, by determining a continued irritation to the respiratory organs, causes morbid predisposition to unfold itself there,—or disease, elsewhere existing, to throw itself upon the lungs. Thus in the very individuals, who in their youth had been scrofulous, but then escaped,—perhaps even recovered from tubercular disease *by means of* repeated catarrh and its sequelæ, bronchial dilatation and pulmonary emphysema,—we find, in the decline of life, catarrh productive of tubercular development, and even of phthisis. We have already seen what an ascendancy gout exercises over catarrh. In like manner hemorrhoidal disease,—which at the age of manhood is prone to manifest its periodical exacerbations by hemorrhage from the rectum,—sometimes gives rise, under the influence of catarrhal irritation, to repeated bronchial hemorrhage. In females, at the period of menstrual decline, catarrh probably leads to similar accidents." P. 269.

After pointing out the various causes inducing those very serious and fatal forms of angina termed œdematosa, Hasse observes that it was probably in consequence of Bayle having noticed only the latent forms depending on mechanical obstruction to the circulation, from long-standing disease in the contiguous parts, and where the œdema is the result of exhaustion, diminished vascular and nervous energy, rather than of inflammation, that this pathologist characterised œdema of the glottis as essentially uninflammatory. The disease is comparatively rare, and our readers may therefore be glad to have Hasse's account of the post-mortem appearances.

"The whole of the mucous membrane between the root of the tongue and the glottis, is uniformly tumefied, so that the outlines of the epiglottis and arytenoid cartilages, together with the numerous folds and recesses in the vicinity of those parts, have become effaced.

"This is the result of inflammatory effusion into the interspaces of the loose cellular texture, subjacent to the mucous membrane. In proportion to the intensity and duration of the inflammatory process, this exudation is sometimes of a purely serous and liquid nature, so as to flow away upon incision; sometimes blended with coagulable materials, and jelly-like; sometimes again mingled in various proportions with pus; sometimes wholly purulent. Hence the tumour, which is always soft, lax, and tremulous, like jelly, varies greatly in colour, being of a pale or of a reddish yellow,—sometimes of a dingy yellow or grayish white, and more or less opaque,—but for the most part superficially dotted with red. The swelling being dependent upon infiltration of the submucous cellular texture, cannot extend to the inferior surface of the epiglottis, because, there, no layer of cellular tissue exists; hence the epiglottis has the aspect of having both its lateral edges bent over towards its nether surface, so as to form a narrow perpendicular groove, which is sometimes almost covered by the overhanging tumour. Neither does the swelling extend to the vocal ligaments (so that the term *œdema glottidis* is not strictly correct); but the tumour, hanging down on each side and in size often exceeding a pigeon's egg, overlays the glottis in such wise as to leave but a narrow opening towards the posterior part, which allows the column of air to pass out during expiration, but is closed up by any attempt at inspiration. In no example recorded did the œdematous infiltration beneath the mucous membrane show itself in any marked degree beyond the glottis. The inner surface of the larynx was reddened, puffy, and covered with a puriform mucous layer. Galen's (Morgagni's) ventricles were always more or less implicated in the submucous tumefaction. The mucous membrane of the affected parts is always variously changed. Its surface is rough, its epithelium partly thrown off in single scales and tufts, or raised by serous fluid; it exhibits here and there blood-specks, or scattered bright-red vascular streaks; it is moreover easily separable, and, when separated, friable. The neighbouring muscles, especially the arytenoid, are sometimes unaltered, at other times saturated in like manner with the effused fluid, in which case they have become blanched, or undergone yellowish or grayish softening. Where the disease is of catarrhal origin, the fauces and the palatine region are generally found simultaneously inflamed, and the tonsils destroyed by suppuration. The inflammation commonly ends where the pharynx passes into the œsophagus, and again at the glottis; yet there have been instances (see Bouillaud's first case) of its pervading the whole of the air-passages and attacking portions of the lungs.

"Œdema of the glottis, when at all violent, commonly proves fatal. Inflammation of the epiglottis *alone* is perhaps more susceptible of cure, in which case the organ appears to undergo hardening, and to shrivel, and diminish in size." P. 273.

In the chapter on Exudative Inflammation of the Air-passages there is one passage having reference to croup which deserves notice. It is observed that, where the false membrane cannot be entirely expelled, the whole mass sometimes liquifies into a grayish or puriform mucus; or else it becomes attenuated and perforated with little holes, without separating, so as occasionally to remain long fastened like a network to the surface of the mucous membrane. In such cases, suffocation may ensue (as it is known to do) some days after apparent amelioration, owing to remnants of the false membrane becoming detached, and clogging up the aperture of the glottis.

The catarrhal pneumonia of Hasse is the peripneumonia notha of the ancients. The condition of the lung in this form of pneumonia is thus described.

"In many instances this variety differs from ordinary pneumonia in nothing else than in the peculiar mode in which it is developed and diffused. In other cases, on the contrary, there is a wide difference in the condition of the pulmonary texture, in the quality of the inflammatory product, and in the phases which the latter undergoes. When near the surface of the lung, the diseased portions rather sink in than bulge out, and are, if recent, somewhat incompressible, but of progressively increasing flabbiness where the disease has been protracted. They are, at the outset, of a dingy, blue reddish hue, which, however, becomes paler by degrees, changing first to a brown-red, and eventually to a yellow-brown. The texture thus altered is altogether impermeable, and soft, though less friable than in hepatization. At the beginning, it is saturated with a turbid reddish fluid, which contains numerous blood-globules; by-and-bye it becomes more and more opaque, gradually passing into grayish,—as though it were mixed with purulent mucus, and ultimately assuming altogether the aspect of a pale, yellowish-brown, puriform fluid. This, when examined by the microscope, is seldom found to be amorpho-granular; it usually contains granule-cells in predominant numbers, but often intermingled with exudation-cells, and sometimes with a proportion of pus-globules. When a large portion of a pulmonary lobe becomes thus disorganized, which is, however rare, it is found shrivelled, lax, moist, of a yellow-brown hue, and wholly devoid of air,—resembling a wet rag. This kind of catarrhal pneumonia appears peculiarly calculated to produce obliteration of the pulmonary texture, with permanent exclusion of air therefrom, and consequent general dilatation of the several branchlets of the bronchia engaged." P. 285.

Catarrhal pneumonia invariably originates in a catarrhal affection of the air-passages, is a frequent result of pertussis, and almost invariably present, to a greater or less extent, in fatal cases of bronchitis, more particularly in children. The diagnosis is usually difficult in consequence of its affecting certain patches of the lung, often of small extent, and frequently occupying the centre of a lobe. In certain epidemics, catarrh is peculiarly apt to take on this form, and the typhoid symptoms occurring sometimes rapidly in the course of bronchitis, are frequently attributable to this complication, and the consequent increased impediment to the aeration of the blood. Hasse states that a further distinction between catarrhal and ordinary pneumonia consists in the presence of plastic exudation in the bronchia leading to the hepatised lobules. That there always is a bronchitis of the tubes in immediate connexion with the affected lobules is certainly true, but we much doubt whether the exudation is always, or even generally, of a plastic character. The very peculiarities of the pneumonia would be, in our opinion, an argument against this statement of Hasse. But the special characters of this affection, both in a pathological and therapeutical point of view, are deserving of further investigation.

Dilatation of the bronchi Hasse describes as of three forms, the first consisting of a single spherical or pouch-like protrusion of the walls of the tube, the second constituted by a series of these cystic dilatations, and the third of distinct character, formed by the uniform dilatation of a number of tubes throughout their whole length, making the portion of lung to which they go appear to consist exclusively of tubes widened to many times their natural size. The former two, or spherical, forms are produced chiefly by bronchitis and tubercular disease of the lungs. The influence of bronchitis is probably exerted in the following way:—

"First, the air-passages are stripped of their epithelium-lining in the ordinary manner, their canals becoming loaded in part with a mucous secretion, in part plugged with fibrinous exudation. This latter occurrence takes place chiefly within certain of the lesser twigs, occasioning a collapse of the adjunct air-cells. The space thus set free is sought to be filled up by expansion of the neighbouring parts, giving rise in the majority of cases to emphysema; where, however, the collapse does not occur close beneath the surface of the lung, but at a greater depth and near a larger bronchial tube, and where it comprehends a larger tract of pulmonary substance, the result is bronchiectasis. These circumstances do not, however, suffice for the formation of a bronchial cavity; the parietes of the involved bronchial tube must needs have previously suffered the changes pointed out by Stokes, namely, loss, through inflammation, of elasticity in the longitudinal, and of contractile power in the annular fibres, with consequent incapacity on the part of either to resist the mechanical influence of forcible inspiration, or of violent cough. It is difficult to say whether Stokes is right in believing that a saccular protrusion of the mucous membrane is caused by yielding of the fibres;—such, however, may probably be the case, where the dilatation is one-sided, and its principal portion external to the axis of the bronchial tube. The analogy, likewise adverted to by Stokes, with the forms of aneurism, must, on the other hand, fall to the ground, as untenable." P. 297.

We see no reason, however, for dissenting from the opinion of Stokes in reference to the analogy between these bronchial and aneurismal dilatations; on the contrary, it affords, we think, the most plausible explanation. With regard to the third variety, or the cylindrical dilatations, Hasse adopts a similar theory to that of Corrigan, who has termed the disease cirrhosis of the lung.

The cylindrical form of bronchiectasis arises where the pulmonary cells have become extensively obliterated by previous pneumonia, and the bronchial tubes been constrained by the pressure of the air to fill up the space vacated, before the parietes of the thorax have had sufficient time to collapse. In like manner, pleurisy may give rise to dilatation, where the effusion is of a character to keep the pulmonary cells long compressed, without subsequently affording them an opportunity for due expansion. "The bronchial tubes not being similarly encumbered, are the more liable to yield to the pressure of the air inspired." If, in addition, the parietes of the air-passages have lost somewhat of their elasticity from the previous inflammation, this passive (as Hasse terms it) dilatation will be the more likely to occur. We doubt, however, whether the dilatation occurring in these circumstances is so entirely a passive phænomenon as Hasse supposes. Why should not the air-cells give way rather than the bronchial tubes, unless indeed it be assumed, that this is prevented by their being filled with inflammatory products, in which case there is no space vacated, and requiring to be supplied by the distended bronchia? We are rather inclined, with Dr. Corrigan, to believe that contraction of the surrounding tissue is an important, if not the main, cause of the distension of the bronchia. We have looked in vain in Gross's work for any explanation of this or any other form of dilated bronchi.

*Emphysema of the Lung.*—Hasse is disposed to coincide with the majority of writers who have followed Laennec in assigning chronic catarrh as the main cause of the most important and frequent variety, viz. that which consists in dilated air-cells.

"My own observation," he says, "of the entire course, the duration, and the general character of the disease, induces me, to a certain extent, to agree with the majority. Catarrh is, without doubt, the principal occasional cause of emphysema; and hooping-cough, in particular, is evidently capable, within a very short space of time, of effecting the highest degree of dilatation in the pulmonary cells. The character and real import of emphysema must, however, be admitted to depend on other causes, as yet not thoroughly made out." P. 304.

"It is, on the whole, surprising how rapidly dilatation of the pulmonary cells may take place, although it requires time to attain such a height as to manifest itself by physical signs. The means whereby catarrh effects a dilatation of the cells, are purely mechanical. In catarrh, the lesser bronchial twigs are obstructed, partly by tenacious mucus, and partly by the swollen mucous membrane. During inspiration, the action of the muscles is powerful enough to impel the air through these obstacles: expiration, which is carried on rather through the force of elasticity than through the medium of the comparatively feeble expiratory muscles, is less complete. Thus more air is inhaled than exhaled, and the surplus, expanding through the natural warmth, ultimately overcomes the resistance due to the elasticity of the pulmonary texture. On the other hand, the more forcible acts of expiration—coughing for example—will rather compel the surrounding vesicles to yield to the pressure of the dilated ones, than allow the air, contained within the latter, to surmount the obstacle to its natural exit. In like manner, swellings of the bronchial glands, &c., or tubercular deposits within the lungs, may give rise to emphysema, by the compression of bronchial tubes,—as shown by Reynaud, Andral, Carswell, and others." P. 305.

These mechanical causes are doubtless efficient to a certain extent, and in a certain number of cases. But the undoubted hereditary origin of the disease in a large proportion of cases, as first clearly shewn by Jackson, and since amply confirmed, together with the history of the disease, in many instances where no such hereditary tendency can be discovered, and an accurate estimate of the structural changes attending the disease, have long since satisfied us that, in the large majority of cases, some original weakness or defect in the walls of the air-cells, and the structure of the lungs generally, is the true and main source of the disease. In other cases, probably, there is an induced weakness of the walls of the pulmonary cells, the result of catarrhal or other previous disease. The account which Gross gives of the disease is meagre and defective in the extreme. After strangely asserting that the specific gravity of an emphysematous lung is "increased!" he goes on to observe, "*vesicular emphysema is rarely observed before the age of fifty!*" The whole of his observations, on its pathological relations and modes of origin, are contained in the following brief passage. "It is a very common attendant on tubercles, aneurismal tumours of the heart and aorta, enlargements of the bronchial glands and asthmatic disorders. Amongst the occasional causes may be enumerated whatever has a tendency to over-distend the air-cells of the lungs, as playing upon wind-instruments, singing, and *loud screaming!*" (P. 440.) Considering that loud screaming is to be reckoned among the occasional causes, we should scarcely have expected the statement that it is "rarely observed before the age of fifty." No allusion whatever is made to the labours of his distinguished and lamented countryman Jackson, although he alludes to Louis, whose deductions were mainly drawn from the data collected by his pupil Jackson.

In remarking on the peculiar shape of the thorax in aggravated cases, Hasse observes, "Louis mentions another kind of projection, or rather filling up, of the supra-clavicular region, which gives a plumpness, he says, to the otherwise emaciated neck. I believe this appearance to have been present in the cases noted by that accurate observer, but have never myself witnessed it. Indeed, I have mostly found, above the vaulting of the chest, and above the clavicles, a decided pit or depression, wherein are displayed, almost like thick, tense cords, the cervical muscles of inspiration, which in general play a subordinate part, but here, where the operation of the principal inspiratory muscles is defective, become prominently active." P. 310.

We can ourselves bear testimony to the accuracy of Hasse's statement, having directed particular attention to this physical sign, and never, in a single instance, met with the filling up of the supra-clavicular region of which Louis speaks, but, on the contrary, with Dr. Stokes, have found the triangular spaces answering to the insertion of the sterno-mastoid and scaleni muscles singularly deep.

The chapter on Tuberculosis of the Lung is carefully compiled and full of instruction. We have, however, so recently brought this subject before the attention of our readers, that it is the less necessary to enter into any detailed examination of Hasse's views. Moreover, as the researches of Mr. Addison and Mr. Rainey have appeared since the publication of Hasse's work, he has been precluded from availing himself of the observations of these gentlemen, which are of great interest and importance in connexion with the minute anatomy of tubercle. We cannot, however, here avoid alluding briefly to the very careful observations of Mr. Rainey,

That gentleman has, we think, incontrovertibly shewn the truth of the opinion of Carswell and others (and adopted by Hasse), that the common opaque yellow tubercle is thrown out into the cavities of the air-cells. In proportion as these become distended by the accumulating tubercular matter, the inter-cellular vascular plexuses become compressed, obliterated, and eventually destroyed, together with the walls of the air-cells. In this way several cells are thrown into one, several tubercles become amalgamated into an irregular mass, penetrated here and there by the crescentic edges of the partially destroyed cell-walls, and on careful examination, presenting occasionally detached fragments of the remains of the inter-cellular plexuses and their corresponding cell-walls. We have thus two important causes of derangement of the respiratory function. Not only is the supply of blood to one part of the lung cut off by the obliteration of its vessels, but also an almost necessary congestion of adjoining portions induced, and thus a tendency to hæmoptysis established.

There cannot, we think, be any doubt that Mr. Rainey's views afford the true explanation of those appearances observed in tubercles which have induced many to believe them to be vascular. It is strong confirmation of these views, that the vessels which are not unfrequently seen, appear, for the most part, as arcs of circles, of the same radius as the plexuses between the air-cells, and of which they are the unabsorbed fragments. The following passage from the Postcript to Mr. Rainey's paper in the last volume of the Medico-Chirurgical Transactions, is so important in connexion with the general pathology of tubercle, that we shall be excused for extracting it. A rabbit, whose lungs, liver, kidney, mesentery, and other parts presented numerous tubercles, all of a scrofulous character, was injected with fine injection.

"Some parts of the lungs were studded with white masses of different sizes; others, even as much as a third of a lobe, appeared very much like a lung which had never respired. On examining the latter, I perceived, in the arterial trunks leading to those parts, distinct masses of white granular matter mixed with the injection; and, continuing the examination, I found that this appearance was due to all the capillaries being literally choked up with this same matter. The air-cells were free from it and contained air. The white masses in the other parts, appeared to be produced by the vessels being filled with this matter, as in the preceding, and also by its escape into the air-cells and surrounding structures. On examining the kidney I found that the vessels were filled in the same manner as in the lungs. I mentioned this to Mr. Quekett, who told me that he had, in scrofulous cases, seen strumous matter mixed with blood which had been pressed out from an artery going to a diseased part."

Hasse justifies himself for classing glanders with affections of the respiratory organs, in consequence of the disease originally and chiefly implicating the nostrils and air-passages. In our present state of comparative ignorance on this subject, the classification may be admitted. Hasse, however, has nothing new to offer on the subject, and it is not even mentioned by Gross.

From his own experience as well as that of others, Hasse has attempted to draw up such an historical account of cancerous tumours in the respiratory organs as shall assist in the detection of the disease during life. The account though very succinct, is valuable and instructive.

"Cancer of the lung is incomparably more frequent in males than in females. Out of 22 cases, collected by myself, 5 concern women, and 17 men. In childhood, the disease is unknown. Of the above 22 cases, 9 occurred between the 20th and 29th years; 8 between the 30th and 39th; 2 between the 40th and 49th; 2 between the 50th and 59th; and 1 between the 70th and 79th years. The morbid disposition is, accordingly, greatest in the prime of life." P. 370.

In almost every instance the tumours in the lungs were medullary, he having only once met with the colloid variety. The medullary cancer was chiefly found in isolated masses; and in the form of infiltration, only where the lungs were the part originally affected, and primary infiltration appears to be characterised by one lung being exclusively involved, the other remaining exempt. From the statistics collected by Dr. Walshe, it would appear that the right lung is the one affected in two-thirds of the cases. These points are of practical importance in reference to the diagnosis. It is an important fact, that cancer in organs whose veins are tributary to the portal system, does not appear to spread to the lungs, although it is known very often to lead to corresponding disease in the liver. This limitation of the disease to the capillary range of the portal vein, Hasse observes, is the more remarkable when we consider the promptness with which medullary cancer, in particular, proceeds from one cluster of lymphatic glands to others situated in the remotest parts of the body. It is also remarkable that the lung shews no disposition to disseminate the morbific elements of which it is the seat; and in the case of primary cancer of the lungs there does not appear to be a single well-marked instance of other internal organs becoming secondarily affected. Cancerous disease of the lungs never co-exists with scrophulous tubercles.

The remaining Sections of Hasse's work, on cysts in the respiratory organs, pseudo melanosis, and diseases of the thymus and thyroid glands, our limits compel us to pass over. We have already given a sufficiently full abstract of the work to enable our readers to judge of its character, and we trust to justify the opinion we have expressed of its merits. For the purposes of reference the book would have been much improved by a more methodical division of the Chapters and Sections.

With Professor Gross's work we need not longer occupy the time of our readers, as it is not likely ever to obtain a place in the literature of this country, where we have so many much better works. The coloured drawings and engravings with which it is illustrated are so coarse as to be for the most part quite useless and altogether beneath criticism.

